# A novel model to label delirium in an intensive care unit from clinician actions

**DOI:** 10.1186/s12911-021-01461-6

**Published:** 2021-03-09

**Authors:** Caitlin E. Coombes, Kevin R. Coombes, Naleef Fareed

**Affiliations:** 1 College of Medicine, The Ohio State University, Columbus, OH 43210 USA; 2 Department of Biomedical Informatics, The Ohio State University College of Medicine, 460 Medical Center Dr., 512 Institute of Behavioral Medicine Research, Columbus, OH 43210 USA; 3 Center for the Advancement of Team Science, Analytics, and Systems Thinking, College of Medicine, The Ohio State University, Columbus, OH 43210 USA

**Keywords:** Delirium, Electronic health records, Intensive care unit, Predictive model, Risk factors

## Abstract

**Background:**

In the intensive care unit (ICU), delirium is a common, acute, confusional state associated with high risk for short- and long-term morbidity and mortality. Machine learning (ML) has promise to address research priorities and improve delirium outcomes. However, due to clinical and billing conventions, delirium is often inconsistently or incompletely labeled in electronic health record (EHR) datasets. Here, we identify clinical actions abstracted from clinical guidelines in electronic health records (EHR) data that indicate risk of delirium among intensive care unit (ICU) patients. We develop a novel prediction model to label patients with delirium based on a large data set and assess model performance.

**Methods:**

EHR data on 48,451 admissions from 2001 to 2012, available through Medical Information Mart for Intensive Care-III database (MIMIC-III), was used to identify features to develop our prediction models. Five binary ML classification models (Logistic Regression; Classification and Regression Trees; Random Forests; Naïve Bayes; and Support Vector Machines) were fit and ranked by Area Under the Curve (AUC) scores. We compared our best model with two models previously proposed in the literature for goodness of fit, precision, and through biological validation.

**Results:**

Our best performing model with threshold reclassification for predicting delirium was based on a multiple logistic regression using the 31 clinical actions (AUC 0.83). Our model out performed other proposed models by biological validation on clinically meaningful, delirium-associated outcomes.

**Conclusions:**

Hurdles in identifying accurate labels in large-scale datasets limit clinical applications of ML in delirium. We developed a novel labeling model for delirium in the ICU using a large, public data set. By using guideline-directed clinical actions independent from risk factors, treatments, and outcomes as model predictors, our classifier could be used as a delirium label for future clinically targeted models.

**Supplementary Information:**

The online version contains supplementary material available at 10.1186/s12911-021-01461-6.

## Background

Delirium is an acute, confusional state associated with a fluctuating disturbance in awareness and cognition arising alongside serious illness [1]. In the intensive care unit (ICU), delirium affects up to 41–50% of patients overall [2, 3], up to 82% of patients with prolonged ICU length of stay (LOS) [3], and over 75% of patients undergoing mechanical ventilation [4]. Patients with in-hospital delirium are at risk for adverse short- and long-term outcomes, including increased LOS, discharge to postacute nursing facilities [3, 5–7], slowed surgical recovery [8], persistent cognitive impairment [9], incident dementia [10], and death [10].

Delirium poses challenges for both researchers and clinicians from incompletely understood pathophysiology [3, 5], multifactorial etiology [3, 11], terminological inconsistency [5], and under-recognition and inappropriate management in the clinical setting [3, 5]. The clinical presentation of the syndrome is broad, including an agitated, hyperactive subtype; a somnolent, hypoactive subtype; or mixed features [5]. The hypoactive subtype is less frequently diagnosed and has poorer prognosis [5]. Additional patients may manifest with subsyndromal delirium or “attenuated delirium syndrome”: a subclinical confusional state meeting part, but not all, of the DSM-5 criteria for delirium [12]. Due in part to delirium’s comorbid presentation with serious illness, advanced age, depression, and dementia[5, 12] and its heterogeneous and fluctuating symptom presentation [12], delirium is often under-recognized in the hospital [5, 12, 13]. Because delirium arises comorbidly, the primary treatment is identification, diagnosis, and treatment of the etiologic organic illness or toxic insult, accompanied by pharmacological and nonpharmacological delirium symptom management [11]. These challenges make delirium an important target of machine learning (ML) [14–22].

Training ML models require a valid delirium label which can accurately capture a patient with the condition. For a method of labeling to be useful as a foundation for clinical prediction, it must be independent of both risk factors and outcomes of interest. Although the gold standard is a provider-administered screening tool such as the Confusion Assessment Method for the ICU (CAM-ICU) [13, 23], these labor-intensive identifiers must be prospectively administered and are not available in all settings [13, 20–22], revealing a need for a delirium identifier that can be abstracted retrospectively and computationally from the medical record.

Two preliminary studies on small cohorts (< 400 patients) have proposed other simple, chart-based labels when CAM-ICU is absent. Kim et al.[24] used the CAM-ICU and provider interview as the gold standard to label delirium with modest sensitivity (30%), high specificity (97%) and high positive predictive value (PPV = 83%) from the presence of either an International Classification of Diseases (ICD) code or antipsychotics use, with improved sensitivity for delirium that was hyperactive or mixed type (64%) or severe (73%). By chart review, Puelle et al.[25] identified eight key words or phrases (altered mental status, delirium, disoriented, hallucination, confusion, reorient, disorient and encephalopathy) with high PPV (60–100%) for delirium (model sensitivity and specificity not reported).

Here we present an assessment of three methods to label delirium in the chart from medical record events. We propose a supervised binary classifier based on counts of 31 clinician actions, including medications, orders, and clinical impressions in free-text notes. All 31 predictors are independent of risk factors and outcomes of interest, generating a labeling method that could be used as a foundation for downstream clinical predictions. We compare this model to Kim et al.’s classification based on ICD code and antipsychotics use (“Kim’s classifier”) and to Puelle et al.’s eight words with high PPV (“Puelle’s classifier”). To the best of our knowledge, we are the first to test these proposals on a large-scale dataset. Because our dataset is too large to permit chart review and CAM-ICU is unavailable, we set ICD code as our initial delirium identifier. We assess the quality of classification of each model by biological validation[26] on clinically meaningful, delirium-associated outcomes, demonstrating superior performance with our model of 31 clinician actions. Our model has the potential to be generalized and implemented across ICU datasets to support improved labeling for downstream clinical predictive modeling.

### Strategies to label and validate delirium in large-scale datasets

In 2015, Inouye et al. proposed research priorities for delirium, including improved diagnosis and subtyping, stratification of high risk patients, biomarker detection, and identification of genetic determinants [3]. Researchers have since applied unsupervised ML, including clustering[15] and latent class analysis [14], to subtype patients. More commonly, supervised ML is used to predict delirium incidence within an ICU stay based on a priori risk factors [21], heart rate variability [17], or medical record events from the first 24 h of hospitalization [16, 18, 20, 27].

To make clinically actionable predictions, the researcher requires a delirium label that is independent of the clinical covariates and predictors of interest. The preferred measures in clinical practice for labeling delirium are nurse- or provider-administered, validated screening tools, including the CAM-ICU[13, 23] and the Intensive Care Delirium Screening Checklist (ICDSC) [13, 28, 29]. CAM-ICU administered during treatment is a mainstay label of delirium in the ML research setting [14–19]. However, variations in institutional practice and physician buy-in can lead to inconsistent use of the CAM or ICDSC in the clinical setting [13]. When CAM-ICU is unavailable or suspect, researchers may employ nurse chart review [20, 21]. However, chart review relies on clinical judgment[25] and poses time and labor costs that grow prohibitive as data sets increase in size.

Other researchers have used ICD codes as a delirium label [22]. Though convenient, ICD codes, especially secondary codes (such as delirium in a critical illness setting), are prone to high levels of missingness and inaccuracy [30–32]. Although the prevalence of delirium in the ICU has been estimated to be as high as 24–82% [2–4], published models have been built using ICD code labels for delirium that may be as sparse as 3.1% [22]. This mismatch between proportion of expected patients with delirium and available ICD codes suggests a risk of outcome misclassification if ICD codes are used, with potential for serious bias in learned model outputs [33]. Weaknesses in delirium labeling underlying much state-of-the-art research calls the generalizability and clinical utility of these studies into question.

Various tools are available when binary outcome misclassification in a dataset is suspected. Sensitivity analysis can be used to adjust the summary output of a logistic regression model, but it relies heavily on frequency estimates supplied by the researcher’s a priori knowledge of the field, and cannot be learned from the model [33]. For some binary classifiers, outcome misclassification can be addressed by tuning model cut-points based on a priori knowledge or researcher goals for sensitivity or specificity or properties of the receiver operating curve (ROC) to enact a desired reclassification, a core practice in diagnostic test development[34] with applications in supervised model refinement [16].

Assessing outcome reclassification on real data is challenging due to absence of a gold standard. However, the concern is pressing: unless model fit is perfect (sensitivity and specificity = 100%), all binary classification inherently generates some degree of “outcome reclassification,” where members labeled as belonging to one group when entering the model are later predicted to belong to the other group. For clinical regression models, Harrell et al. proposed that the concordance index or c-index, calculated from pairwise comparisons of a prognostic indicator between classified and reclassified subjects, could be employed as a “clinically meaningful” measure of model goodness-of-fit [37]. We have previously proposed the related principle of biological validation: that ML assignments can be meaningfully validated by employing well-understood biological outcomes when ground-truth is unavailable [26]. Inspired by Harrell’s approach, we compare five prognostic measures between classified and reclassified groups to biologically validate outcome reclassification and model goodness-of-fit for delirium identification.

## Methods

### Study population

Study data were drawn from Medical Information Mart for Intensive Care-III (MIMIC-III), a freely available database of electronic health record (EHR) data collected on 63,157 intensive care unit (ICU) admissions at Beth Israel Deaconess Medical Center from 2001 to 2012 [38–41]. Delirium within a hospitalization was defined by ICD-9 code [24]. (Additional file 2: Table A.1) Unique admissions were included for all adult patients ≥ 18 years of age with ICU length-of-stay (LOS) less than 31 days (48,451 hospitalizations). Restricting LOS removed 2,315 outlier hospitalizations (4.6%) with LOS up to 295 days. From the cohort population, 25% of positives and negatives were randomly sampled and reserved for a test set (12,135 admissions), retaining 75% for training (36,406 admissions).

**Table 1:** Definitions of four classified and re-classified categories generated by a binary classifier

Classification group	Has delirium ICD-9 code	Model predicts delirium
Double-Positives	Yes	Yes
Reclassified-Positives	No	Yes
Reclassified-Negatives	Yes	No
Double-Negatives	No	No

For any binary classifier with less than 100% accuracy, model testing results in some degree of reclassification of positives and/or negatives, generating four groups. For example, some admissions with an ICD-9 code for delirium are labeled as negative by the model, leading to re-classification

### A novel model predicting delirium from clinician actions

#### Variable selection

We proposed a model to label presence of delirium in a chart based on clinician actions. We hypothesized that changes in clinical actions concordant with diagnostic work-up for delirium can serve as an indicator that the clinical team had made a delirium diagnosis. Clinician actions presumed to indicate a response to delirium onset were identified from published guidelines for delirium work-up and abstracted from electronic health record (EHR) data. These included 18 laboratory and imaging orders and 4 medications [13, 42]. Pharmacologic interventions were selected based on evidence of widespread use for the management of delirium, not by efficacy or other clinical measures [13]. Clinical impressions were extracted from the presence of eight words or phrases with high PPV for delirium in EHR notes [25]. Additional file 2: Table A.2 lists the 31 included clinical actions. No steps were taken to identify or impute missing values. Occurrence of clinician actions were formed into an event count matrix across each admission [43]. A more detailed description of data pre-processing, with code, is available in Additional file 1: File B.

**Table 2:** Demographic characteristics of a cohort of adult ICU patients

	Total	Patients with Delirium ICD-9 Code	Patients without Delirium ICD-9 Code	p-Value
	n (%)^a^	n (%)^b^	n (%)^b^	
*Total*	48,541	3,850	44,691	–
*Sex*				
Male	27,220 (56.1%)	2,181 (56.6%)	25,039 (56.0%)	0.538^c^
Female	21,321 (43.9%)	1,675 (43.4%)	19,646 (44.0%)	
*Race/Ethnicity*				
White or Caucasian	34,792 (71.7%)	2,857 (74.1%)	31,935 (71.5%)	< 0.005^d^
Black or African	4,668 (9.6%)	381 (9.9%)	4,287 (9.6%)	
Hispanic or Latino	1,720 (3.5%)	129 (3.4%)	1,591 (3.6%)	
Asian	1,133 (2.3%)	74 (1.9%)	1,059 (2.4%)	
Other^e^	6,228 (12.8%)	415 (10.8%)	5,813 (13.0%)	
*Age at Admission*^*7*^				
< 30 years	2,222 (4.6%)	140 (3.6%)	2,082 (4.6%)	< 0.0005^f^
30–39 years	2,568 (5.3%)	164 (4.3%)	2,404 (5.4%)	
40–49 years	5,151 (10.6%)	388 (10.1%)	4,763 (10.7%)	
50–59 years	8,396 (17.3%)	607 (15.7%)	7,789 (17.4%)	
60–69 years	10,117 (20.8%)	715 (18.5%)	9,402 (21.0%)	
70–79 years	10,042 (20.7%)	789 (20.5%)	9,253 (20.7%)	
80–89 years	7,432 (15.3%)	747 (19.4%)	6,685 (15.0%)	
≥ 90 years	2,613 (5.4%)	306 (7.9%)	2,307 (5.2%)	
*Length of Stay*^g^				
< 5 days	17,406 (35.9%)	783 (20.3%)	16,623 (37.2%)	< 0.0005^f^
5 – 10 days	19,131 (39.4%)	1,470 (38.1%)	17,661 (39.5%)	
11 – 20 days	9,163 (18.9%)	1,149 (29.8%)	8,014 (17.9%)	
21 – 30 days	2,841 (5.9%)	454 (11.8%)	2,387 (5.3%)	

Differences between patients with and without delirium ICD-9 codes were tested with t-tests and chi-squared tests, as appropriate

^a^Percent of total patients

^b^Percent within subgroup (with or without delirium ICD-9 code)

^c^Pearson’s Chi-squared test with Yates’ continuity correction

^d^Pearson’s Chi-squared test

^e^Other race, race unavailable, multi race ethnicity (0.21% of total cohort), Native American, Native Hawaiian, or Pacific Islander (0.06% of cohort)

^f^Welch’s two-sample t-test

^g^Continuous variable in the MIMIC-III discretized for illustration in this table

#### Supervised model selection and refinement

We compared performance of five binary ML classifiers [16, 17, 19, 22], including logistic regression (stats R-package), Classification and Regression Trees (CART; rpart R-package) [44, 45], supervised random forests (randomForest) [46, 47], naïve Bayes (e1071) [48, 49], and support vector machines (SVM; e1071) [49, 50]. (Additional file 1: File A.1) The logistic regression model underwent refinement and feature selection by stepwise forwards and backwards selection, L1/LASSO (Least Absolute Shrinkage and Selection Operator) penalization [51, 52], L2/Ridge penalization [53], and combined L1-L2 penalization (penalized). [54] Model performance on the training set was compared by ROC visualization and AUC (pROC) [55]. (Additional file 1: File A.2) The top performing model was selected by maximum AUC. Model development is reported here in accordance with Transparent Reporting of a multivariable prediction model for Individual Prognosis or Diagnosis (TRIPOD) guidelines [56].

#### Reclassification and binary threshold determination

Logistic regression generates a model with a log-odds threshold set at zero to divide hospitalizations with incident delirium from those without. This “natural” or “default” cut-point reflects the prior probability of delirium within the cohort, and is therefore susceptible to error from outdated prior information (such as known misclassification). As commonly implemented in diagnostic test development, we tuned the cut-point of our binary classifier to calibrate sensitivity and specificity to correct for known misclassification [34], a technique in practice in delirium supervised model development [16]. Because we suspect ICD-9 code missingness [30–32], we desire a model with high sensitivity. In the case of known misclassification, we believe that some of the additional positives generated by increased sensitivity represent true, but unlabeled, positives that have been reclassified. These reclassified positives represent hospitalizations containing real incident delirium, but lacking ICD-9 codes due to a priori outcome misclassification from known ICD-9 code missingness [30–32]. Thus, reclassification by up-tuning sensitivity allows us to generate a model that better labels the presence of true delirium.

On training data, we compared six algorithmic methods for reclassification of a binary model by tuning sensitivity: the Youden index [57], maximizing both sensitivity and specificity, maximizing accuracy, minimizing the distance to ROC (0,1), maximizing accuracy given a minimum constraint of sensitivity, and maximizing sensitivity given a minimal specificity constraint (Additional file 1: A.3; cutpointr R-package) [58]. We determined the threshold of choice based on concordance between measures, choosing a cut-point that represented trends between tuning methods. We also visualized reclassification by each cut-point by density plot.

The final model was trained on training data using the binary classifier with highest AUC, selected by maximum AUC, and the cut-point with highest measured concordance. This best-performing model was run on retained test data. Validation was performed on test data only.

### Comparison models

We identified two related models in the literature proposed from chart review to predict incidence of delirium within a hospital stay from clinician actions and implemented them at an expanded scale.

To assess Puelle’s classifier [25], we trained a logistic regression model with eight binary predictors for presence or absence at any point in a hospitalization of eight words in notes with high PPV for delirium on the training set (Additional file 1: Material A.4.1). Previously, we had implemented the same eight words in our model of 31 clinician actions (Additional file 2: A.2). We omitted Puelle’s final criterion, “’alert and oriented’ (< 3)” due to difficulty of abstracting this data point from free-text note fields without natural language processing. The resultant model was validated on the test set. The binary threshold was chosen with the Youden Index. We compared our novel model to Puelle’s classifier by the Akaike Information Criterion (AIC) and the Bayes Information Criterion (BIC) [59].

We tested Kim’s classifier [24] by labeling hospitalizations as delirium-positive if they contained a delirium ICD-9 code or if anti-psychotics were prescribed at any point during hospitalization (Additional file 1: Material A.4.2). Admissions were delirium-negative if a delirium ICD-9 code was not applied and anti-psychotics were not administered. This simple recategorization did not require training and was applied directly to the test set.

### Validation of reclassified models by clinical markers and outcomes

Statistical measures of final model performance included sensitivity, specificity, PPV, negative predictive value (NPV), AUC (for supervised models), and comparison against expected prevalence of ICU delirium.

Reclassification was validated on five clinically meaningful demographic and outcome measures: age at admission [3], discharge location [5–7], death in hospital, death within 30 days of admission [38], and one-year mortality from admission [10]. To assess success and meaningfulness of re-classification and goodness-of-fit for each model, we separated admissions into four groups (Table 1). First, we compared ICD-Positives and Double-Negatives. If these were significantly different, we report tests comparing ICD-Positives to Reclassified-Positives, Double-Negatives to Reclassified-Negatives, and Reclassified-Positives to Reclassified-Negatives. Similarity or difference between groups was assessed using Tukey multiple comparisons of means for continuous data and Pearson’s chi-squared tests with pairwise comparisons with the Holm correction for categorical data [60, 61].

## Results

From 48,451 unique adult admissions in MIMIC-III with LOS ≤ 31 days, we identified 3,850 patients with delirium by ICD-9 codes (7.9%). Demographic characteristics and pertinent outcomes of the cohort are described in Table 2. Briefly, the group with patients with delirium had statistically significant differences with the group without delirium for race/ethnicity, age at admission, and length of stay.

### Novel model of 31 clinician actions

**Fig. 1:**
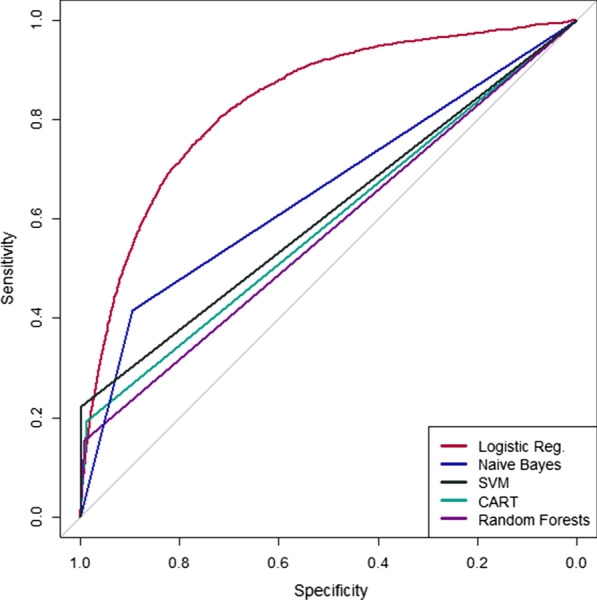
Comparison of ROC curves for 5 binary classifiers for presence or absence of delirium in the critical care setting. By AUC, logistic regression (unadjusted odds) outperformed supervised binary classification by naïve Bayes, support vector machines (SVM), Classification and Regression Trees (CART), and random forests

Figure 1 summarizes the performance of five supervised binary classifiers by ROC. Logistic regression performed best on the training set (AUC = 0.83). Naïve Bayes, SVM, CART, and random forests produced models with AUC of 0.66, 0.61, 0.59, and 0.58, respectively. Attempts to refine the logistic regression with forwards and backwards selection (AUC = 0.83), L1 (LASSO) penalization (AUC = 0.83), L2 (Ridge) penalization (AUC = 0.83), or combined L1 and L2 penalization (AUC = 0.83) did not improve performance. Of 31 clinical actions in the base model, forwards and backwards selection retained 25 predictors. L1, L2, and combined L1-L2 penalization retained all 31 clinical actions. Because three of four feature selection methods recommended inclusion of all 31 features and the potential for knowledge loss with predictor elimination, the model with 31 clinical actions was selected.

Table 3 presents 17 highly significant predictors (p < 0.001) from the final, multiple logistic regression model of 31 clinical actions. The full model can be found in Additional file 2: Table A.3. Among clinical impressions captured from single words in text notes, odds of delirium were higher with each note mentioning “mental status” (OR = 1.14), “deliri*”(OR = 1.12), “hallucin*”(OR = 1.25), or “confus*” (OR = 1.16), and “disorient*”(OR = 1.10). Odds of delirium were lower for each note mentioning “reorient*” (OR = 0.86). Among laboratory tests, odds of delirium were significantly greater with clinical orders for urine culture (OR = 1.13), thyroid function test (OR = 1.12), serum B12 or folate (OR = 1.45), and blood or urine toxicology screen (OR = 1.28). Prescription orders for antipsychotics (OR = 1.44), benzodiazepines (OR = 1.08), and dexmedetomidine (OR = 1.43) were associated with higher odds of delirium.

**Table 3:** Highly significant predictors from a multiple logistic regression model to classify delirium in the medical record

	Odds Ratio	95% CI Lower Bound	95% CI Upper Bound	Z-Value	p-Value
“Mental status”	1.144	1.114	1.176	9.766	< 0.000005
“Deliri*”	1.121	1.082	1.163	6.249	< 0.000005
“Hallucin*”	1.252	1.161	1.351	5.820	< 0.000005
“Confus*”	1.160	1.123	1.199	8.872	< 0.000005
“Reorient*”	0.863	0.807	0.923	− 4.270	0.00002
Urine culture	1.131	1.084	1.179	5.682	< 0.000005
ABG^a^	0.978	0.972	0.984	− 7.097	< 0.000005
Renal function panel	1.041	1.024	1.058	4.871	< 0.000005
CBC^b^	0.965	0.952	0.978	− 5.186	< 0.000005
Thyroid function test	1.122	1.058	1.189	3.872	0.00011
Toxicology screen	1.275	1.217	1.336	10.243	< 0.000005
Autoimmune serology	0.408	0.292	0.556	− 5.464	< 0.000005
B vitamins	1.451	1.315	1.598	7.477	< 0.000005
HIV antibody	0.479	0.314	0.705	− 3.574	0.00035
Antipsychotics	1.443	1.400	1.488	23.589	< 0.000005
Benzodiazepines	1.076	1.048	1.103	5.614	< 0.000005
Dexmedetomidine	1.432	1.260	1.626	5.513	< 0.000005

Presented here are 17 predictors from 31 clinical actions from a multiple logistic regression model with p < 0.001. Coefficients and confidence intervals are presented for odds. The full model is available in Additional file 2: A.3

^a^Arterial blood gas

^b^Complete blood count

### Reclassification and model comparison

**Fig. 2:**
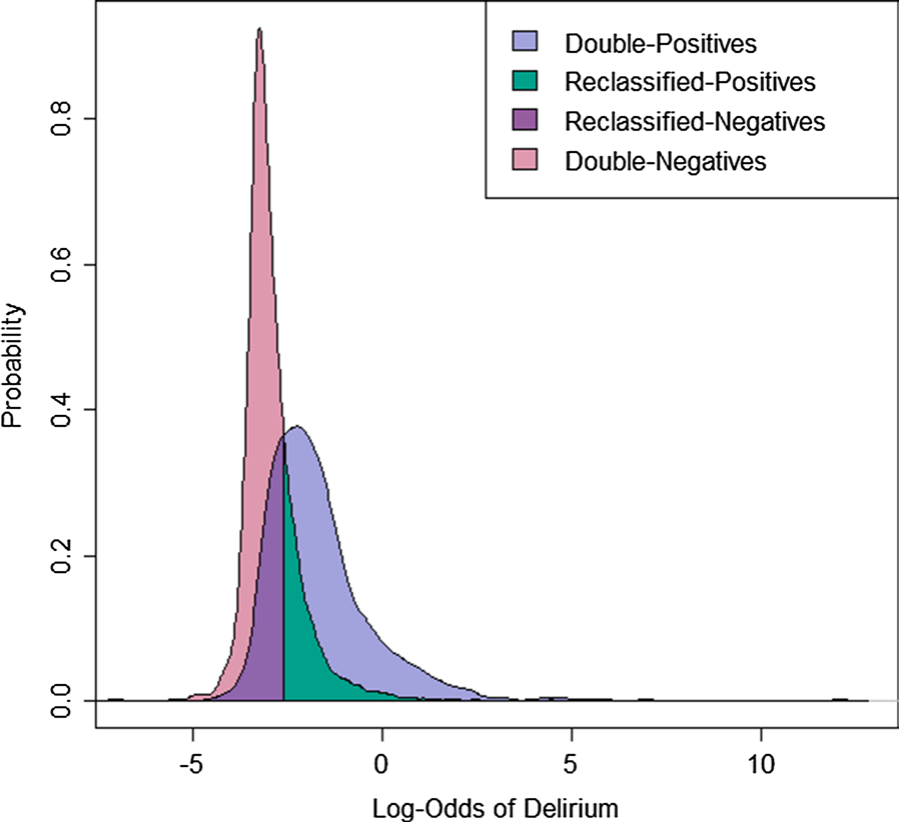
Probability density plot of four reclassification groups generated by our model predicting delirium from 31 clinical actions. Binary classification by multiple logistic regression generates four groups, including two groups of reclassified hospitalizations

We compared six metrics for sensitivity (Se) tuning: the Youden Index (Se = 80%), maximizing sensitivity and specificity (Se = 80%), maximizing accuracy (Se = 5.20%), minimizing the distance to ROC (0,1) (Se = 76%), maximizing accuracy constraining sensitivity (Se = 50%), and maximizing sensitivity constraining specificity (Se = 92%). Additional file 2: Table A.4 illustrates the cut-point, sensitivity, specificity, and accuracy of six methods for tuning a threshold for a binary logistic classifier. Figure 2 visualizes reclassification of the test cohort by our model into four groups (ICD-Positives, Reclassified-Positives, Reclassified-Negatives, Double-Negatives) along our chosen method, the Youden Index. (Additional file 2: Figure A.1 presents this visualization for Puelle’s classifier.)

**Table 4:** Significant differences for three models between four reclassification groups on five clinical measures

	Reclassification Groups			
	Double + vs Double─	Double + vs Reclassified +	Reclassified + vs Reclassified─	Double- vs Reclassified─
Expected Relationship	Different p < 0.05^a^	Same p > 0.05^a^	Different p < 0.05^a^	Same p > 0.05^a^
*Novel Model*				
Age	< 0.00005	< 0.00005	< 0.00005	< 0.00005
Discharge location	< 0.00005	< 0.00005	< 0.00005	< 0.00005
Death in hospital	< 0.00005	0.00011	0.0452	0.0859
30-day mortality	0.00072	0.115	0.00017	0.0011
one-year mortality	< 0.00005	0.178	0.0017	< 0.00005
*Puelle’s Classifier*				
Age	< 0.00005	0.00097	< 0.00005	0.964
Discharge location	< 0.00005	< 0.00005	0.00062	0.00062
Death in hospital	0.0017	< 0.00005	0.820	0.103
30-day mortality^b^	0.0949	–	–	–
one-year mortality	< 0.00005	< 0.00005	0.660	0.0015
*Kim’s Classifier*^c^				
Age	< 0.00005	0.0130	–	–
Discharge location	< 0.00005	< 0.00005	–	–
Death in hospital	0.0035	< 0.00005	–	–
30-day mortality^b^	0.472	–	–	–
one-year mortality	< 0.00005	0.0010	–	–

Binary classification generates four groups of subjects, including two groups of reclassified hospitalizations, which were compared by biological validation to assess model goodness-of-fit. Double-Positives are expected to differ from Double-Negatives. In the case of successful reclassification, we expect Double-Positives to be similar to Reclassified-Positives, Double-Negatives to be similar to Reclassified-Negatives, and Reclassified-Positives to differ from Reclassified-Negatives. Bolded fields represent p-values concordant with expectations of good model fit

^a^p-values were generated from pairwise chi-squared testing with the Holm correction for all measures except age, which was tested with pairwise Tukey multiple comparisons of means

^b^Pairwise testing was not performed in the event of no significant difference between Double-Positives and Double-Negatives

^c^The Kim model does not generate reclassified negatives, making associated tests unavailable

On the test data, logistic regression with threshold reclassification by the Youden Index (cut-point = −2.72) and all 31 clinical predictors included resulted in a model with AUC of 0.83, 79.4% sensitivity, 71.5% specificity, 19.7% PPV, and 97.6% NPV, evaluated against delirium ICD-9 codes. This threshold reclassification suggests a delirium prevalence within the ICU cohort up to 32.5%. Puelle’s classifier, using a Youden Index cut-point of -2.671, produced 79.8% sensitivity, 72.2% specificity, 19.8% PPV, and 97.6% NPV, predicting a cohort delirium prevalence 31.9%. Puelle’s classifier had AIC of 18,378 and BIC of 18,455. Our novel model had AIC of 16,786 and BIC of 17,058. By definition, Kim’s reclassification categorized all ICD-Positives as having delirium and did not generate Reclassified-Negatives, resulting in 100% sensitivity, 85.7% specificity, 37.7% PPV, and 100% NPV, with an estimated cohort prevalence of delirium up to 21.1%.

Table 4 illustrates similarity and difference between four reclassification groups on five clinical measures. With Kim’s reclassifier, Double-Positives and Double-Negatives differed significantly for age, discharge location, death during hospitalization, and one-year mortality, but did not differ for 30-day mortality (p = 0.472). Double-Positives and Reclassified-Negatives differed significantly in all tested categories. Under Puelle’s classifier, Double-Positives and ICD-Negatives differed significantly in all clinical validation measures except 30-day mortality (p = 0.949). Reclassified-Negatives and Double-Negatives did not significantly differ for age (p = 0.964) and death in hospital (p = 0.103). However, ICD-Positive and Reclassified-Positives differed significantly on all tested validation measures. Reclassified-Positives and Reclassified-Negatives did not differ significantly by death in hospital (p = 0.820) or one-year mortality (0.660). In our novel model, Double-Positives and Double-Negatives differed significantly on all five validation measures. Double-Positives and Reclassified-Positives did not significantly differ by 30-day mortality (p = 0.115) or one-year mortality (p = 0.178). Double-Negatives and Reclassified-Negatives did not differ significantly by death in hospital. Reclassified-Positives and Reclassified-Negatives differed significantly (p < 0.05) for all 5 validation measures.

## Discussion

ML holds the potential to unlock improved diagnosis, risk stratification, and treatment of delirium in the ICU, a complex syndrome associated with serious morbidity and mortality. Before ML can be used to make clinically actionable predictions, informaticians developing models for delirium incidence, prognosis, and treatment need tools to accurately label patients with delirium in large datasets, despite serious flaws with current labeling methods. Ideally, delirium researchers need a valid, efficient, computational tool that is independent of clinical variable of interest to label patients with delirium in large datasets without the need for chart review on in-person clinical assessments. A high-accuracy, computationally-generated label could be used for training future models on pressing clinical questions, including identifying timing of delirium onset in the hospital course or classifying patients with delirium into clinically relevant clusters. Here, we proposed to label delirium from clinician actions, using placement of orders associated with standard workup of delirium as a surrogate for clinicians recognizing delirium in real time.

After comparison of five supervised ML methods and four methods of feature selection, we proposed a novel, multiple logistic regression model to label ICU delirium from counts of 31 clinician actions abstracted from clinical guidelines, with high AUC (0.83). If predictors are not independent, we expect improved performance from non-linear models. However, because these 31 clinical actions are regularly employed in wider clinical practice independent of delirium and thus none are specific for delirium, it is possible that a greater than expected independence between covariates resulted in unexpectedly good performance from the logistic model. The assumption of independence is reinforced by a correlation matrix with less than 4% of 31 predictors having a Spearman’s *ρ* of ≥ 0.6. The logistic model is both appropriate to the data and offers clearer, biological interpretability than many non-linear models.

Model performance on a training set was validated on a randomly selected test set. The model was concordant with clinical intuition, with odds of delirium higher with words such as “deliri*,” “hallucin*,” and “disorient*,” but odds of delirium lower with “reorient*.” Marked elevations in odds of delirium were associated with toxicology screening, used to detect delirium from substance intoxication or withdrawal, and prescription of antipsychotics or dexmedetomidine. Evidence of intoxication falls within the DSM-5 criteria for diagnosis of delirium [1, 12]. Guidelines recommend antipsychotics as the drug class of choice for symptomatic treatment of delirium [13]. Dexmedetomidine is recommended as a preferred drug for management of delirium on mechanically ventilated patients [13].

We compared our labeling model to two similar models previously proposed in the literature to abstract delirium incidence from chart review. Both our model and Puelle’s classifier produced sensitivity and specificity between 71 and 80%, indicating good fidelity to delirium ICD-9 codes with modest reclassification of both positives and negatives. Although the implementation of Puelle’s classifier has similar PPV and sensitivity with fewer predictors, our novel model had superior performance by both lower AIC and BIC.

Kim et al. [24] reported low sensitivity (30%) but high specificity (97%) of their classifier on a prospective study of 184 adults. Specificity on the expanded MIMIC-III data set was 85.7%. Our implementation of Kim classifier never generates reclassified negatives: all patients with ICD-9 codes for delirium are classified in the delirium group by definition. Thus, the 100% sensitivity and 100% NPV reflect definitions for model creation, not quality of fit. The PPV of Kim’s classifier (37.7%) surpasses that of Puelle’s classifier (19.8%) and our model (19.7%). However, PPV is also defined by simple re-categorization in Kim’s classifier, and is not indicative of improved performance. For both Kim’s and Puelle’s classifiers, reduced performance with computational application on the expanded, MIMIC-III dataset suggest limitations in generalizability and validation of these small-scale proposals.

Because ground-truth is not reasonably attainable in these data by chart review due to their very large size, we compared goodness-of-fit of the three models by biological validation [26]. First, we assume that, for a good model, predicted prevalence of delirium (sum of ICD-Positives and Reclassified-Positives) should approach known ICU delirium prevalence from the literature. In a meta-analysis of 48 studies on ICU delirium, Krewulak et al. [2] obtained an overall pooled delirium prevalence of 31%. Kim’s classifier predicted delirium prevalence above ICD-9 code frequency (21.1%). Our model (32.5%) and Puelle’s classifier (31.9%) predicted delirium prevalence concordant with Krewulak’s pooled figures, indicating an appropriate quantity of reclassified patients.

We further biologically validate against clinically meaningful outcome measures. We compared classification and reclassification groups by age, discharge location, short-term risk of death, and one-year mortality. Our method of model validation rests on the principle that application of any binary classifier that does not have perfect (100%) sensitivity and specificity reclassifies subjects, such that some number of subjects receive a classification from the model that differs from their input label assignment (Table 1, Fig. 2). If the binary classification model is valid, then this unavoidable reclassification should result in reclassified subjects resembling their reclassified assignment more so than their label assignment across the five comparison measures. On the basis of biological validation, our novel model markedly outperformed Kim’s and Puelle’s classifiers, correctly capturing significant differences between Double-Positives and Double-Negatives and between Reclassified-Positives and Reclassified-Negatives on all five measures. Delirium is a heterogeneous syndrome with subtype variation, including an under-diagnosed hypoactive subtype and a subclinical form [5, 12]. Thus, differences between Double-Positives and Reclassified-Positives may represent variability in clinician practice between delirium subtypes, with our model reclassifying patients belonging to subtypes underrepresented in previous studies.

### Limitations

The clinical utility of our novel model rests on important contextual factors. First, our study is based on publicly available data from one institution. However, our model uses one of the largest count of observations for developing a ML model for delirium than previously used in other studies. Although we propose the implementation of a generalizable labeling model that is relatively less labor intensive than models that depend upon screening tools, ICD codes, and chart review (many of which that are not easily available), we recognize the importance of heterogeneity that will exist at both an institutional and a local provider level [62]. Examples include sub-group and temporal considerations and idiosyncratic coding and documentation practices. There is a need for local validation and recalibration to ensure the optimal performance of our labeling method [63]. Because of under-identification of hypoactive or milder delirium in the clinical[5] or analytic[24] setting, deviations in model goodness of fit may reflect variation in clinical practice and patient presentation between delirium subtypes.

As noted previously, our model’s overall performance, albeit relatively better than other counterpart models, still has constraints in terms of factors such as sensitivity and PPV. Like other ML models, decisions to implement our model will require considerations about tradeoffs around model performance factors, the costs of model implementation, and the implications of false-positives [64, 65]. The potential response to positive cases and other approaches that can be used to establish true-positive cases will be critical. Finally, because this model does not use time-dependent variables, it may not be able to label a patient with delirium until after all encounter data is available.

Future work to predict delirium subtypes from the medical record is warranted. Patients being presented with other diseases, example SARS-CoV-2, may result in the introduction of other features that may improve the calibration of the model given the prevalence of such a disease in the local ICU. ICU delirium has been shown to be comorbid with SARS-CoV-2, arising from disorientation and social isolation, use of mechanical ventilation, and an aging patient population [66].

## Conclusions

We developed a novel labeling model for delirium in the ICU using a large data set from a publicly available database. This database has been previously used to develop ML models for other applications [67, 68]. Our model incorporates 31 clinical actions as features, an approach that has been previously overlooked in other delirium prediction models. We assessed the performance of our labeling model based on other delirium prediction models and biological markers of significance. Our model demonstrates relative superiority based on the assessment rubric; however, more validation and recalibration are needed to consider important contextual factors that may arise before and during the use of the model in a local ICU. These results provide a tool to aid future researchers developing ML classifiers for ICU patients with delirium.

## Supplementary Information

**Additional file 1**. Study model development and model comparison tables.

**Additional file 2**. Study R Markdown file with data pre-processing and variable selection.

## Data Availability

The dataset supporting the conclusions of this article is available in the MIMIC-III repository,[38, 39, 41] https://mimic.physionet.org/.

## Abbreviations

AIC: Akaike information criterion
AUC: Area under the receiver operating characteristic curve
BIC: Bayes information criterion
CAM-ICU: Confusion assessment method
CART: Classification and regression trees
DSM: Diagnostic and statistical manual of mental disorders, 5th Edition
EHR: Electronic health record
ICU: Intensive care unit
ICD: International classification of diseases
ICDSC: Intensive care delirium screening checklist
LASSO: Least absolute shrinkage and selection operator
LOS: Length of stay
ML: Machine learning
MIMIC: Medical information mart for intensive care
PPV: Positive predictive value
PU: Positive unlabeled
SARS-CoV-2: Severe acute respiratory syndrome coronavirus 2
TRIPOD: Transparent reporting of a multivariable prediction model for individual prognosis or diagnosis
NPV: Negative predictive value
OR: Odds ratio
