# Supplementary material for: A novel model to label delirium in an intensive care unit from clinician actions
Source: BMC Med Inform Decis Mak. 2021 Mar 9;21:97. doi: 10.1186/s12911-021-01461-6 (PMC7941123; doi:10.1186/s12911-021-01461-6)
Supplement: Supplementary file 1 — Additional file 1. Study model development and model comparison tables. [file 12911_2021_1461_MOESM1_ESM.docx]

---

title: "Supplementary Material B: Data Pre-Processing and Variable Selection"

date: "`r Sys.Date()`"

output: html_document

---

# 1. Variable Selection from MIMIC-III

MIMIC-III was built locally as a PostGresSQL 10 database and queried from RStudio to construct the data set for analyses.

```{r con}

library(odbc)

con <- DBI::dbConnect(odbc::odbc(),

Driver = "{PostgreSQL Unicode(x64)}",

Server = "localhost",

Database = "mimic"

# ...

)

```

```{sql searchPath, connection=con}

set search_path to mimiciii

```

** Note: Data from MIMIC-III were queried separately for hospitalizations with and without ICD-9 codes of interest. For these two groups, identical code was run for variable selection and pre-processing. For brevity, this code is presented only once in sections 1.1-1.5 of this document.**

## 1.1 Prescriptions

For patients with delirium ICD-9 codes:

```{sql rawScripts, connection=con, output.var="rawScripts"}

SELECT admissions.hadm_id,

prescriptions.drug, prescriptions.drug_name_generic, prescriptions.drug_name_poe,

prescriptions.startdate

FROM admissions

LEFT JOIN diagnoses_icd ON admissions.hadm_id = diagnoses_icd.hadm_id

LEFT JOIN prescriptions ON admissions.hadm_id = prescriptions.hadm_id

WHERE diagnoses_icd.icd9_code IN ('2930', '2931', '29281', '29011', '2903', '29041', '2910', '2939', '78009', '29381', '29382', '29383', '29384', '29389', '29012', '29013', '29043', '29211', '29212', '2922', '78002', '2902', '29042', '2908', '2909', '2920', '29282', '3483', '34831', '34839', '34982', '78097')

```

For patients without delirium ICD-9 codes:

```{sql rawScripts2, connection=con, output.var="rawScripts2"}

SELECT admissions.hadm_id,

prescriptions.drug, prescriptions.drug_name_generic, prescriptions.drug_name_poe,

prescriptions.startdate

FROM admissions

LEFT JOIN diagnoses_icd ON admissions.hadm_id = diagnoses_icd.hadm_id

LEFT JOIN prescriptions ON admissions.hadm_id = prescriptions.hadm_id

WHERE diagnoses_icd.icd9_code NOT IN ('2930', '2931', '29281', '29011', '2903', '29041', '2910', '2939', '78009', '29381', '29382', '29383', '29384', '29389', '29012', '29013', '29043', '29211', '29212', '2922', '78002', '2902', '29042', '2908', '2909', '2920', '29282', '3483', '34831', '34839', '34982', '78097')

```

All benzodiazepines in MIMIC-III were identified to all terminate in the suffix "azepam." A list of antipsychotics was obtained from Up to Date on March, 2020. We searched for each drug within the MIMIC-III database for inclusion.

```{r}

scr <- rawScripts

# Benzodiazepines

bz <- grep("azepam", scr$drug)

scr$DrugType <- NA

scr[bz, "DrugType"] <- "Benzodiazepines"

# Antipsychotics

ap <- c(grep("Chlorproma", scr$drug),

grep("Fluphena", scr$drug),

grep("Haloperid", scr$drug),

grep("Loxapi", scr$drug),

grep("Perphenaz", scr$drug),

grep("Pimoz", scr$drug),

grep("Thiroida", scr$drug),

grep("Thiotix", scr$drug),

grep("Trifluoper", scr$drug),

grep("Aripipr", scr$drug),

grep("Asenap", scr$drug),

grep("Brexipip", scr$drug),

grep("Caripra", scr$drug),

grep("Clozap", scr$drug),

grep("Iloperi", scr$drug),

grep("Lurasi", scr$drug),

grep("Olanzap", scr$drug),

grep("Paliperi", scr$drug),

grep("Pimavan", scr$drug),

grep("Quetiap", scr$drug),

grep("Resperid", scr$drug),

grep("Ziprasi", scr$drug)

)

scr[ap, "DrugType"] <- "AntiPsychotics"

# Rivastigmine

scr[grep("Rivastig", scr$drug), "DrugType"] <- "Rivastigmine"

# Dexmedetomidine

scr[grep("Dexmede", scr$drug), "DrugType"] <- "Dexmedetomidine"

scripts <- scr[!is.na(scr$DrugType),]

```

## 1.2 Procedures: Imaging and EKG

Our initial attempt was to abstract procedures and orders, including orders for labs and microbiology, from dated CPT codes. Finding that CPT codes in MIMIC-III are limited and, although a date field is present, dates are most commonly NA, we abstracted labs, procedures, orders, and microbiology from other tables within the databaste.

We drew radiology and ECG data from the NOTEVENTS table. When selecting imaging studies for inclusion, we reviewed the list of all studies included in MIMIC-III and retained all radiologic stuies that met delirium work-up guidelines.

### Radiology

Radiology events can be abstracted with timestamp from the NOTEVENTS table.

```{sql radiology, connection=con, output.var="radiology"}

SELECT admissions.hadm_id,

noteevents.cgid, noteevents.chartdate,

noteevents.category, noteevents.description

FROM admissions

LEFT JOIN diagnoses_icd ON admissions.hadm_id = diagnoses_icd.hadm_id

LEFT JOIN noteevents ON admissions.hadm_id = noteevents.hadm_id

WHERE noteevents.category = 'Radiology'

AND diagnoses_icd.icd9_code IN ('2930', '2931', '29281', '29011', '2903',

'29041', '2910', '2939', '78009', '29381', '29382', '29383', '29384',

'29389', '29012', '29013', '29043', '29211', '29212', '2922', '78002',

'2902', '29042', '2908', '2909', '2920', '29282', '3483', '34831',

'34839', '34982', '78097')

```

```{r rads}

# Chest X-ray

vchest <- c("CHEST (PA, LAT & OBLIQUES)" , "CHEST (SINGLE VIEW)", "CHEST (APICAL LORDOTIC ONLY)" , "R RIB UNILAT, W/ AP CHEST RIGHT" , "CHEST AP ONLY" , "RIB BILAT, W/AP CHEST" , "AP/PA SINGLE VIEW EXPIRATORY CHEST" , "L RIB UNILAT, W/ AP CHEST LEFT" , "L CHEST (LAT DECUB ONLY) LEFT", "L CHEST (LAT DECUB ONLY) LEFT" , "CHEST (LAT DECUB ONLY)" , "B CHEST (LAT DECUB ONLY) BILAT", "R CHEST (LAT DECUB ONLY) RIGHT", "CHEST (PA & LAT)" , "CHEST (PORTABLE AP)" )

# Brain Imaging

vhead <- c("MR HEAD W & W/O CONTRAST" , "BRAIN SCAN" , "CT HEAD W/O CONTRAST", "MR HEAD W/O CONTRAST", "CT EMERGENCY HEAD W/O CONTRAST", "CT HEAD W/ CONTRAST", "CT HEAD W/ & W/O CONTRAST", "MR HEAD W/ CONTRAST", "CT HEAD W/ ANESTHESIA W/ CONTRAST", "PORTABLE HEAD CT W/O CONTRAST")

v <- c(vchest, vhead)

radiology <- radiology[radiology$description %in% v,]

radiology$RadType <- ifelse(radiology$description %in% vchest, "CXR", "BrainImaging")

```

### ECG

ECG events can be abstracted with timestamp from the NOTEVENTS table.

```{sql ecg, connection=con, output.var="ecg"}

SELECT admissions.hadm_id,

noteevents.cgid, noteevents.chartdate,

noteevents.category, noteevents.description

FROM admissions

LEFT JOIN diagnoses_icd ON admissions.hadm_id = diagnoses_icd.hadm_id

LEFT JOIN noteevents ON admissions.hadm_id = noteevents.hadm_id

WHERE noteevents.category = 'ECG'

AND diagnoses_icd.icd9_code IN ('2930', '2931', '29281', '29011', '2903',

'29041', '2910', '2939', '78009', '29381', '29382', '29383', '29384',

'29389', '29012', '29013', '29043', '29211', '29212', '2922', '78002',

'2902', '29042', '2908', '2909', '2920', '29282', '3483', '34831',

'34839', '34982', '78097')

```

### The Procedures Table

```{r procTab1}

ecg$ProcType <- ecg$category

colnames(radiology)[colnames(radiology) == "RadType"] <- "ProcType"

procedures <- rbind(radiology, ecg)

```

## 1.3 Labs and Microbiology

Evidence of laboratory events or microbiology was determined from presence of a result of a test in MIMIC-III. Lab values were not utilized in further analysis. The complement of laboratory tests available in MIMIC-III is restricted. When selecting laboratory and microbiology tests for inclusion, we reviewed the list of all labs included in MIMIC-III and retained all labs that met delirium work-up guidelines. For laboratory tests conducted in panels (such as complete blood count or renal function panel), the laboratory test was counted as the panel if at least one of the tests included in the panel was performed.

### Laboratory Events

```{sql rawLabs, connection=con, output.var="rawLabs"}

SELECT admissions.hadm_id,

d_labitems.label, d_labitems.fluid, d_labitems.category,

labevents.charttime, labevents.value, labevents.valuenum

FROM admissions

LEFT JOIN diagnoses_icd ON admissions.hadm_id = diagnoses_icd.hadm_id

LEFT JOIN labevents

INNER JOIN d_labitems ON labevents.itemid = d_labitems.itemid

ON admissions.hadm_id = labevents.hadm_id

WHERE diagnoses_icd.icd9_code IN ('2930', '2931', '29281', '29011', '2903', '29041', '2910', '2939', '78009', '29381', '29382', '29383', '29384', '29389', '29012', '29013', '29043', '29211', '29212', '2922', '78002', '2902', '29042', '2908', '2909', '2920', '29282', '3483', '34831', '34839', '34982', '78097')

```

```{r}

labs <- rawLabs

# Arterial Blood Gas

labs$LabType[labs$category == c("BLOOD GAS", "Blood Gas")] <- "ABG"

# Complete Blood Count

cbc <- c("Red Blood Cells", "White Blood Cells", "RBC", "WBC", "Platelet Count", "Basophils", "Neutrophils", "Lymphocytes", "Monocytes", "Bands", "Macrocytes", "Absolute Lymphocyte Count", "Lymphocytes, Percent", "Reticulocyte Count, Automated", "Granulocyte Count", "Macrophages", "WBC Count", "Eosinophil Count", "Monocyte Count")

labs$LabType[labs$label %in% cbc] <- "CBC"

# Electrolyte Panel with Calcium, Magnesium, Phosphorous

elec <- c("Calcium, Total", "Magnesium", "Phosphate", "Potassium", "Sodium", "Free Calcium")

labs$LabType[labs$label %in% elec] <- "ElectrolytePanel"

# Liver Function, including Albumin

liver <- c("Alanine Aminotransferase (ALT)", "Albumin", "Asparate Aminotransferase (AST)", "<Albumin>")

labs$LabType[labs$label %in% liver] <- "LiverFunction"

# Renal Function, including BUN, Creatinine

renal <- c("Creatinine", "Urea Nitrogen", "Estimated GFR (MDRD equation)", "Creatinine Clearance", "Creatinine, Serum", "Urine Creatinine", "24 hr Creatinine", "Albumin/Creatinine, Urine", "Alkaline Phosphatase")

labs$LabType[labs$label %in% renal] <- "RenalFunction"

# Toxicology Screen

tox <- c("Barbiturate Screen", "Tricyclic Antidepressant Screen", "Barbiturate Screen, Urine", "Cocaine, Urine", "Opiate Screen, Urine", "Acetaminophen", "Benzodiazepine Screen", "Salicylate", "Amphetamine Screen, Urine", "Benzodiazepine Screen, Urine", "Methadone, Urine", "Marijuana")

labs$LabType[labs$label %in% tox] <- "ToxScreen"

# Vitamin B12 & Folate

# Note: Thiamine/B1 is not available in data set.

b <- c("Vitamin B12", "Folate")

labs$LabType[labs$label %in% b] <- "Bvitamins"

# Thyroid Function (TSH, T4)

t4 <- c("Thyroxine (T4), Free", "Thyroid Stimulating Hormone", "Thyroxine (T4)")

labs$LabType[labs$label %in% t4] <- "ThyroidFunction"

# Cortisol

labs$LabType[labs$label == "Cortisol"] <- "Cortisol"

# Ammonia

labs$LabType[labs$label == "Ammonia"] <- "Ammonia"

# Sedimentation Rate

labs$LabType[labs$label == "Sedimentation Rate"] <- "SedimentationRate"

# Autoimmune Serologies (ANA, c-ANCA, p-ANCA)

ai <- c("Anti-Nuclear Antibody, Titer", "Anti-Nuclear Antibody", "Anti-Neutrophil Cytoplasmic Antibody")

labs$LabType[labs$label %in% ai] <- "AutoimmuneSerology"

# HIV antibody

labs$LabType[labs$label == "HIV Antibody"] <- "HIVantibody"

# Lumbar Puncture (as Laboratory Test)

lp <- c("Total Protein, CSF", "WBC, CSF", "Glucose, CSF", "RBC, CSF", "Hematocrit, CSF", "Miscellaneous, CSF")

labs$LabType[labs$label %in% lp] <- "LumbarPunctureLabs"

laboratory <- labs[!is.na(labs$LabType),]

```

### Microbiology Events

```{sql rawMicro, connection=con, output.var="microbiology"}

SELECT admissions.hadm_id,

microbiologyevents.chartdate, microbiologyevents.charttime,

microbiologyevents.spec_itemid, microbiologyevents.spec_type_desc,

microbiologyevents.org_name

FROM admissions

LEFT JOIN diagnoses_icd ON admissions.hadm_id = diagnoses_icd.hadm_id

LEFT JOIN microbiologyevents

ON admissions.hadm_id = microbiologyevents.hadm_id

WHERE diagnoses_icd.icd9_code IN ('2930', '2931', '29281', '29011', '2903', '29041', '2910', '2939', '78009', '29381', '29382', '29383', '29384', '29389', '29012', '29013', '29043', '29211', '29212', '2922', '78002', '2902', '29042', '2908', '2909', '2920', '29282', '3483', '34831', '34839', '34982', '78097')

```

```{r}

# Blood cultures

vblood <- c("BLOOD CULTURE", "SEROLOGY/BLOOD", "Blood (Toxo)", "Blood (EBV)", "Blood (CMV AB)")

# Urine cultures

vurine <- c("URINE", "URINE,SUPRAPUBIC ASPIRATE", "URINE,PROSTATIC MASSAGE", "URINE,KIDNEY")

# Lumbar puncture:

lp <- "CSF;SPINAL FLUID"

# Subset and Clean

# generate the simplified LabType

mi <- microbiology

mi <- mi[mi$spec_type_desc %in% c(vblood, vurine, lp),]

mi$LabType[mi$spec_type_desc %in% vblood] <- "BloodCulture"

mi$LabType[mi$spec_type_desc %in% vurine] <- "UrineCulture"

mi$LabType[mi$spec_type_desc %in% lp] <- "LumbarPuncture"

#remove unnecessary information

mi$spec_itemid <- NULL

mi$org_name <- NULL

#remove duplicates

dups <- duplicated(mi[,c(1,2,5)])

mi <- mi[!dups,]

microbiology <- mi

```

## 1.4 Words with high PPV for delirium

```{sql rawWords, connection=con, output.var="rawWords"}

SELECT admissions.hadm_id,

noteevents.cgid, noteevents.chartdate,

regexp_matches(noteevents.text, 'AMS', 'i'),

regexp_matches(noteevents.text, 'mental status', 'i'),

regexp_matches(noteevents.text, 'deliri', 'i'),

regexp_matches(noteevents.text, 'hallucin', 'i'),

regexp_matches(noteevents.text, 'confus', 'i'),

regexp_matches(noteevents.text, 'reorient', 'i'),

regexp_matches(noteevents.text, 'disorient', 'i'),

regexp_matches(noteevents.text, 'encephalopathy', 'i')

FROM admissions

LEFT JOIN diagnoses_icd ON admissions.hadm_id = diagnoses_icd.hadm_id

LEFT JOIN noteevents ON admissions.hadm_id = noteevents.hadm_id

WHERE diagnoses_icd.icd9_code IN ('2930', '2931', '29281', '29011', '2903',

'29041', '2910', '2939', '78009', '29381', '29382', '29383', '29384',

'29389', '29012', '29013', '29043', '29211', '29212', '2922', '78002',

'2902', '29042', '2908', '2909', '2920', '29282', '3483', '34831',

'34839', '34982', '78097')

```

```{r}

colnames(rawWords) <- c("hadm_id", "cgid", "chartdate", "ams", "mentalstatus", "deliri", "hallucin", "confus", "reorient", "disorient", "encephalopathy")

```

## 1.5 A table of all events

Using the 5 tables generated in earlier documents (laboratory, micriobiology, procedures, words, prescriptions), we combine them into a single Events table. This process was repeated for both groups (patients with and without delirium ICD-9 codes).

```{r str}

# Procedures

procedures$category <- NULL

procedures$cgid <- NULL

procedures <- procedures[, c(1,4,3,2)]

procedures$timedate <- as.POSIXct(NA)

colnames(procedures) <- c("hadm_id", "event", "details", "date", "timedate")

# Words

ams <- rawWords[,c("hadm_id", "chartdate", "ams")]

ams$ams[!is.na(ams$ams)] <- "AMS"

ams <- ams[!is.na(ams$ams),]

colnames(ams) <- c("hadm_id", "chartdate", "Event")

ms <- rawWords[,c("hadm_id", "chartdate", "mentalstatus")]

ms$mentalstatus[!is.na(ms$mentalstatus)] <- "MentalStatus"

ms <- ms[!is.na(ms$mentalstatus),]

colnames(ms) <- c("hadm_id", "chartdate", "Event")

deliri <- rawWords[,c("hadm_id", "chartdate", "deliri")]

deliri$deliri[!is.na(deliri$deliri)] <- "Deliri"

deliri <- deliri[!is.na(deliri$deliri),]

colnames(deliri) <- c("hadm_id", "chartdate", "Event")

hallucin <- rawWords[,c("hadm_id", "chartdate", "hallucin")]

hallucin$hallucin[!is.na(hallucin$hallucin)] <- "Hallucin"

hallucin <- hallucin[!is.na(hallucin$hallucin),]

colnames(hallucin) <- c("hadm_id", "chartdate", "Event")

confus <- rawWords[,c("hadm_id", "chartdate", "confus")]

confus$confus[!is.na(confus$confus)] <- "Confus"

confus <- confus[!is.na(confus$confus),]

colnames(confus) <- c("hadm_id", "chartdate", "Event")

reorient <- rawWords[,c("hadm_id", "chartdate", "reorient")]

reorient$reorient[!is.na(reorient$reorient)] <- "REorient"

reorient <- reorient[!is.na(reorient$reorient),]

colnames(reorient) <- c("hadm_id", "chartdate", "Event")

disorient <- rawWords[,c("hadm_id", "chartdate", "disorient")]

disorient$disorient[!is.na(disorient$disorient)] <- "DISorient"

disorient <- disorient[!is.na(disorient$disorient),]

colnames(disorient) <- c("hadm_id", "chartdate", "Event")

encephalopathy <- rawWords[,c("hadm_id", "chartdate", "encephalopathy")]

encephalopathy$encephalopathy[!is.na(encephalopathy$encephalopathy)] <- "Encephalopathy"

encephalopathy <- encephalopathy[!is.na(encephalopathy$encephalopathy),]

colnames(encephalopathy) <- c("hadm_id", "chartdate", "Event")

words <- rbind(ams, ms, deliri, hallucin, confus, reorient, disorient, encephalopathy)

words$details <- NA

words$timedate <- as.POSIXct(NA)

words <- words[,c(1,3,4,2,5)]

colnames(words) <- c("hadm_id", "event", "details", "date", "timedate")

# microbiology

microbiology <- microbiology[,c(1,5,4,2,3)]

colnames(microbiology) <- c("hadm_id", "event", "details", "date", "timedate")

# laboratory

laboratory$fluid <- NULL

laboratory$value <- NULL

laboratory$valuenum <- NULL

laboratory$category <- NULL

laboratory$date <- as.POSIXct(NA)

laboratory <- laboratory[, c(1,4,2,5,3)]

colnames(laboratory) <- c("hadm_id", "event", "details", "date", "timedate")

# prescriptions

scripts$drug_name_generic <- NULL

scripts$drug_name_poe <- NULL

scripts <- scripts[, c(1,4,2,3)]

colnames(scripts) <- c("hadm_id", "event", "details", "date")

scripts$timedate <- as.POSIXct(NA)

# join all 5 tables

fullevents <- rbind(procedures, words, microbiology, laboratory, scripts)

```

### Data Cleaning

We remove duplicates by removing extra descriptive information about each event and standardizing to a single timedate field, to generate a clean, longform dataset.

```{r}

# remove duplicates

events <- fullevents

events$details <- NULL

dups <- duplicated(events)

events <- events[!dups,]

# join time and date to 1 field

e <- events

# collapse to 1 date field

for(i in 1:length(e$timedate)){

if(is.na(e$timedate[i])){

e$timedate[i] <- e$date[i] + 16*60*60

# Set the date from GMT to EDT; add 12 hours to set to noon.

}

}

e$date <- NULL

events <- e

```

# 2. Demographic Data

```{sql rawOutDemo, connection=con, output.var="rawOutDemo"}

SELECT admissions.hadm_id,

admissions.admittime, admissions.dischtime, admissions.deathtime,

admissions.admission_type, admissions.admission_location, admissions.discharge_location,

admissions.insurance, admissions.language, admissions.religion, admissions.marital_status,

admissions.ethnicity, admissions.hospital_expire_flag,

diagnoses_icd.icd9_code,

d_icd_diagnoses.short_title,

patients.subject_id, patients.gender, patients.dob, patients.dod,

icustays.los,

drgcodes.drg_type, drgcodes.drg_code, drgcodes.description,

drgcodes.drg_severity, drgcodes.drg_mortality

FROM admissions

LEFT JOIN diagnoses_icd ON admissions.hadm_id = diagnoses_icd.hadm_id

LEFT JOIN d_icd_diagnoses ON diagnoses_icd.icd9_code = d_icd_diagnoses.icd9_code

LEFT JOIN patients ON admissions.subject_id = patients.subject_id

LEFT JOIN icustays ON admissions.hadm_id = icustays.hadm_id

LEFT JOIN drgcodes ON admissions.hadm_id = drgcodes.hadm_id

```

```{r}

rt <- rawOutDemo

# age

# All dates of birth in MIMIC-III for patients > 89 yo are shifted to comply with HIPAA.

rt$age <- as.numeric(difftime(rt$admittime, rt$dob, units="days"))/365

rt$age[rt$age > 89] <- 90

# length of stay was re-calculated due to missingness in available field

rt$LOS <- as.numeric(difftime(rt$dischtime, rt$admittime, units="days"))

rt$los <- NULL

rt$admission_type <- as.factor(rt$admission_type)

rt$admission_location <- as.factor(rt$admission_location)

rt$discharge_location <- as.factor(rt$discharge_location)

rt$insurance <- as.factor(rt$insurance)

rt$religion <- as.factor(rt$religion)

rt$ethnicity <- as.factor(rt$ethnicity)

rt$gender <- as.factor(rt$gender)

#remove duplicates

rt$icd9_code <- NULL

rt$short_title <- NULL

rt$drg_code <- NULL

rt$drg_mortality <-NULL

rt$drg_severity <- NULL

rt$drg_type <- NULL

rt$description <- NULL

dups <- duplicated(rt)

rt <- rt[!dups,]

demo <- rt

```

```{r}

cs <- demo[, c("hadm_id", "subject_id", "gender", "LOS", "age", "admittime")]

dups <- duplicated(cs)

cs <- cs[!dups,]

cs$admittime <- NULL

```

# 3. Count Data Generation

## Positives (Has ICD-9 Code for Delirium)

```{sql positives, connection=con, output.var="m"}

SELECT admissions.hadm_id,

FROM admissions

WHERE diagnoses_icd.icd9_code IN ('2930', '2931', '29281', '29011', '2903', '29041', '2910', '2939', '78009', '29381', '29382', '29383', '29384', '29389', '29012', '29013', '29043', '29211', '29212', '2922', '78002', '2902', '29042', '2908', '2909', '2920', '29282', '3483', '34831', '34839', '34982', '78097')

```

```{r}

e <- events # for hospitalizations with a delirium ICD-9 code

l <- unique(e$event)

l <- l[l != "LumbarPunctureLabs"]

n <- as.data.frame(matrix(NA, nrow=1, ncol=33))[-1,]

colnames(n) <- c(l, "hadm_id")

# generate counts

h <- unique(e$hadm_id)

length(h)

for(i in 1:length(h)){

m <- e[e$hadm_id == h[i],]

for(j in 1:length(l)){

lj <- l[j]

n[i,j] <- sum(!is.na(m$timedate[m$event == lj]))

}

n[i, "hadm_id"] <- h[i]

}

# restrict to positives

n <- n[n$hadm_id %in% m,]

# restrict to inclusion criteria

n <- n[!(n$hadm_id %in% demo$hadm_id[demo$LOS >= 31]),]

n <- n[!(n$hadm_id %in% demo$hadm_id[demo$age < 18]),]

pos <- n

```

## Negatives (No ICD-9 Code)

```{r}

e <- events # for hospitalizations without ICD-9 code

n <- as.data.frame(matrix(NA, nrow=1, ncol=33))[-1,]

colnames(n) <- c(l, "hadm_id")

# generate counts

h <- unique(e$hadm_id)

length(h)

for(i in 1:length(h)){

m <- e[e$hadm_id == h[i],]

for(j in 1:length(l)){

lj <- l[j]

n[i,j] <- sum(!is.na(m$timedate[m$event == lj]))

}

n[i, "hadm_id"] <- h[i]

}

# restrict to negatives

n <- n[!(n$hadm_id %in% m),]

# restrict to inclusion criteria

n <- n[!(n$hadm_id %in% demo$hadm_id[demo$LOS >= 31]),]

n <- n[!(n$hadm_id %in% demo$hadm_id[demo$age < 18]),]

neg <- n

# bind

# set status (positives are 1, negatives are 0)

neg$status <- 0

pos$status <- 1

n <- rbind(pos, neg)

# lumbar puncture was acquired from MIMIC-III in 2 forms, which are collapsed

n$LumbarPuncture <- n$LumbarPuncture + n$LumbarPunctureLabs

n$LumbarPunctureLabs <- NULL

counts <- n # for a full, joined cohort

```

# 4. Test and Training Sets

Test hospitalizations are randomly selected from the cohort, retaining 25% for test data and 75% for training data. In sampling, 25% of positives and 25% of negatives are set aside for testing, ensuring consistent proportions of positives and negatives in test and training data.

n <- counts

```{r}

counts <- n

hp <- n$hadm_id[n$status==1]

lp <- length(hp)

hn <- n$hadm_id[n$status==0]

ln <- length(hn)

pos_test_id <- sample(hp, 1/4*lp, replace=FALSE) #positives chosen for the test set

neg_test_id <- sample(hn, 1/4*ln, replace=FALSE) #negatives chosen for the test set

test <- n[n$hadm_id %in% c(pos_test_id, neg_test_id),]

training <- n[!(n$hadm_id %in% c(pos_test_id, neg_test_id)),]
