# Supplementary material for: A novel model to label delirium in an intensive care unit from clinician actions
Source: BMC Med Inform Decis Mak. 2021 Mar 9;21:97. doi: 10.1186/s12911-021-01461-6 (PMC7941123; doi:10.1186/s12911-021-01461-6)
Supplement: Supplementary file 2 — Additional file 2. Study R Markdown file with data pre-processing and variable selection. [file 12911_2021_1461_MOESM2_ESM.html]

Supplementary Material A


# Supplementary Material A

#### 2021-02-02

- Table A.1. International Classification of Diseases 9 (ICD-9) diagnosis codes used as indicators of delirium in the critical care setting
- Table A.2 Clinical actions for the workup of delirium
- A.1 Supervised Model Selection
- A.2 Logistic Model Refinement
- Table A.3 Logistic regression model for the binary classification of delirium from 31 clinical actions
- A.3 Sensitivity Tuning
- Table A.4 Cut-point, sensitivity, specificity, and accuracy of 6 methods for tuning a threshold for a binary logistic classifier.
- A.4.1 Comparison Model 1: Puelle’s classifier of 8 words with high PPV
- Supplementary Figure A.1. Probability density plot of 4 reclassification groups generated by Puelle’s classifier
- A.4.2 Comparison Model 2: Kim’s re-classification

*Note: Citation numbers in this document correspond to the bibliography in the main manuscript.*

### Table A.1. International Classification of Diseases 9 (ICD-9) diagnosis codes used as indicators of delirium in the critical care setting

We chose presence or absence of at least one of 32 ICD-9 diagnosis codes previously studied for the identification of delirium as a binary indicator of delirium during an ICU stay.**[Kim 2017]**

| Code | Description |
| --- | --- |
| 290.11 | Presenile dementia with delirium |
| 290.12 | Presenile dementia with delusional features |
| 290.13 | Presenile dementia with depressive features |
| 290.20 | Senile dementia with delusional features |
| 290.30 | Senile dementia with delirium |
| 290.41 | Vascular dementia, with delirium |
| 290.42 | Vascular dementia, with delusions |
| 290.43 | Vascular dementia, with depressed mood |
| 290.80 | Other specified senile psychotic conditions |
| 290.90 | Unspecified senile psychotic condition |
| 291.00 | Alcohol withdrawal delirium |
| 292.00 | Drug withdrawal |
| 292.11 | Drug-induced psychotic disorder with delusions |
| 292.12 | Drug-induced psychotic disorder with hallucinations |
| 292.20 | Pathological drug intoxication |
| 292.81 | Drug-induced delirium |
| 292.82 | Drug-induced persisting dementia |
| 293.00 | Delirium due to conditions classified elsewhere |
| 293.10 | Subacute delirium |
| 293.81 | Psychotic disorder with delusions in conditions classified elsewhere |
| 293.82 | Psychotic disorder with hallucinations in conditions classified elsewhere |
| 293.83 | Mood disorder in conditions classified elsewhere |
| 293.84 | Anxiety disorder in conditions classified elsewhere |
| 293.89 | Other specified transient mental disorders due to conditions classified elsewhere |
| 293.90 | Unspecified transient mental disorder in conditions classified elsewhere |
| 348.30 | Encephalopathy, unspecified |
| 348.31 | Metabolic encephalopathy |
| 348.39 | Other encephalopathy |
| 349.82 | Toxic encephalopathy |
| 780.02 | Transient alteration of awareness |
| 780.09 | Other alteration of consciousness |
| 780.97 | Altered mental status |

### Table A.2 Clinical actions for the workup of delirium

We chose 31 features from clinical guidelines for the diagnostic appraisal and treatment of delirium. We hypothesized that changes in clinical actions to capture care team response to delirium as an indicator that the clinical team had made a delirium diagnosis. Clinical impressions from free-text notes were abstracted from counts of 8 words that are highly sensitive to the presence of delirium in the EHR.**Puelle** Four pharmaceutical interventions were used to indicate initial treatment response.**Barr, Oropello** Diagnostic workup to identify delirium etiology was indicated by presence of 19 diagnostic, imaging, and laboratory tests.**Harrison’s**

| Category | Action |
| --- | --- |
| Clinical Impression | AMS |
| Clinical Impression | mental status |
| Clinical Impression | deliri\* |
| Clinical Impression | hallucin\* |
| Clinical Impression | confus\* |
| Clinical Impression | reorient\* |
| Clinical Impression | disorient\* |
| Clinical Impression | encephalopathy |
| Diagnostic Workup | Complete blood count |
| Diagnostic Workup | Electrolyte panel including calcium, magnesium, phosphorus |
| Diagnostic Workup | Liver function tests, including albumin |
| Diagnostic Workup | Renal function tests, including BUN, creatinine |
| Diagnostic Workup | Urinalysis and culture |
| Diagnostic Workup | Chest radiograph |
| Diagnostic Workup | Blood cultures |
| Diagnostic Workup | Electrocardiogram |
| Diagnostic Workup | Arterial blood gas |
| Diagnostic Workup | Serum and/or urine toxicology screen |
| Diagnostic Workup | Cerebral imaging: CT or MRI, head or brain; without/without contrast material |
| Diagnostic Workup | Lumbar puncture |
| Diagnostic Workup | B Vitamin levels: B12, folate |
| Diagnostic Workup | Thyroid-stimulating hormone (TSH) and free T4 |
| Diagnostic Workup | Cortisol |
| Diagnostic Workup | Serum ammonia |
| Diagnostic Workup | Sedimentation rate |
| Diagnostic Workup | Autoimmune serologies: antinuclear antibodies (ANA), ANCA |
| Diagnostic Workup | HIV antibody |
| Pharmaceuticals | Antipsychotics |
| Pharmaceuticals | Benzodiazepines |
| Pharmaceuticals | Rivastigmine |
| Pharmaceuticals | Dexmedetomidine |

### A.1 Supervised Model Selection

In this and the following section, we provide clean code from our supervised model selection and refinement.

*Note: The MIMIC-III dataset is (publicly available)[https://mimic.physionet.org/], and Supplementary Material B provides our code for data pre-processing. Sections A.1 through A.4 of this document were applied to processed training data, here called “**n**”.*

We tested 5 supervised binary classification algorithms, including logistic regression.

```
model <- glm(status ~ 
                 CXR + BrainImaging + ECG + AMS + MentalStatus + Deliri + Hallucin + Confus + 
                 REorient + DISorient + Encephalopathy + UrineCulture + BloodCulture + LumbarPuncture + 
                 ABG + ElectrolytePanel + RenalFunction + CBC + LiverFunction +
                 ThyroidFunction + Cortisol + ToxScreen + AutoimmuneSerology + Bvitamins + 
                 Ammonia + SedimentationRate + HIVantibody + AntiPsychotics + Benzodiazepines + 
                 Dexmedetomidine + Rivastigmine
       , data=n, family=binomial)
```

For all models, quality was assessed by predicting on training data, visualizing ROC, and calculating AUC.

```
p <- predict(model)
library(pROC)
r <- roc(n$status~p)
plot(r)
auc(r)
```

Binary classifiers assessed included recursive partitioning by Classification and Regression Trees (CART)…

```
library(rpart)
library(rpart.plot)

model <- rpart(as.factor(n$status)~., data=n[,1:31])
rpart.plot(model, roundint=FALSE)

p <- predict(model, data=n[,1:31])
p <- as.data.frame(p)
p$pp <- NA
for(i in 1:length(p$pp)){
  p$pp[i] <- ifelse(p$`1`[i] > p$`0`[i], 1, 0)
}
r <- roc(n$status~p$pp)
plot(r)
auc(r)
```

random forests…

```
library(randomForest)
model <- randomForest(as.factor(n$status)~., data=n[,1:31])
```

naive Bayes…

```
library(e1071)
model <- naiveBayes(as.factor(n$status)~., data=n[,1:31])
```

and support vector machines.

```
library(e1071)
model <- svm(as.factor(n$status)~., data=n[,1:31])
```

### A.2 Logistic Model Refinement

Logistic regression, the top performing model by AUC, was refined. We compared 4 methods of feature selection by model fitting, prediction on training data, ROC, and AUC, as above. Feature selection methods included forwards-and-backwards selection…

```
model2 <- step(model)
```

… penalization by L1 (lasso), L2 (ridge), and mixed L1-L2 logistic regression models. We used the cross-validated log-likelihood to optimize lambdas for L1 and L2 penalized regression (i.e., \(\lambda\_1\) and \(\lambda\_2\)).

For L1 optimization (but not L2), there is the risk that the optimization will converge to a local (as opposed to global) maximum. Before optimizing \(\lambda\_1\), we profiled and plotted to assess presence and number of local vs. global maxima.

```
library(penalized)
fit1 <- profL1(response=n$status, 
                   penalized= ~CXR + BrainImaging + ECG + AMS + MentalStatus + Deliri + Hallucin + Confus + 
                        REorient + DISorient + Encephalopathy + UrineCulture + BloodCulture + LumbarPuncture + 
                        ABG + ElectrolytePanel + RenalFunction + CBC + LiverFunction +
                        ThyroidFunction + Cortisol + ToxScreen + AutoimmuneSerology + Bvitamins + 
                        Ammonia + SedimentationRate + HIVantibody + AntiPsychotics + Benzodiazepines + 
                        Dexmedetomidine + Rivastigmine,
                   data=n[,1:31],
                   model="logistic",
                   fold=10, plot=TRUE)             
opt1 <- optL1(response=n$status, 
                   penalized= ~CXR + BrainImaging + ECG + AMS + MentalStatus + Deliri + Hallucin + Confus + 
                        REorient + DISorient + Encephalopathy + UrineCulture + BloodCulture + LumbarPuncture + 
                        ABG + ElectrolytePanel + RenalFunction + CBC + LiverFunction +
                        ThyroidFunction + Cortisol + ToxScreen + AutoimmuneSerology + Bvitamins + 
                        Ammonia + SedimentationRate + HIVantibody + AntiPsychotics + Benzodiazepines + 
                        Dexmedetomidine + Rivastigmine,
                   data=n[,1:31],
                   model="logistic",
                   fold=fit1$fold)
l1 <- opt1$lambda
l1
```

\(\lambda\_2\) could be optimized without the profiling step.

```
opt2 <- optL2(response=n$status, 
                   penalized= ~CXR + BrainImaging + ECG + AMS + MentalStatus + Deliri + Hallucin + Confus + 
                        REorient + DISorient + Encephalopathy + UrineCulture + BloodCulture + LumbarPuncture + 
                        ABG + ElectrolytePanel + RenalFunction + CBC + LiverFunction +
                        ThyroidFunction + Cortisol + ToxScreen + AutoimmuneSerology + Bvitamins + 
                        Ammonia + SedimentationRate + HIVantibody + AntiPsychotics + Benzodiazepines + 
                        Dexmedetomidine + Rivastigmine,
                   data=n[,1:31],
                   model="logistic",
                   fold=fit1$fold)
l2 <- opt2$lambda
l2
```

We generated 3 models: one with the \(\lambda\_1\) penalty, one with \(\lambda\_2\) penalization, and one with both.

```
modell1 <- penalized(response=n$status, 
                   penalized= ~CXR + BrainImaging + ECG + AMS + MentalStatus + Deliri + Hallucin + Confus + 
                        REorient + DISorient + Encephalopathy + UrineCulture + BloodCulture + LumbarPuncture + 
                        ABG + ElectrolytePanel + RenalFunction + CBC + LiverFunction +
                        ThyroidFunction + Cortisol + ToxScreen + AutoimmuneSerology + Bvitamins + 
                        Ammonia + SedimentationRate + HIVantibody + AntiPsychotics + Benzodiazepines + 
                        Dexmedetomidine + Rivastigmine,
                   data=n[,1:31],
                   lambda1=l1, model="logistic")

modell2 <- penalized(response=n$status, 
                   penalized= ~CXR + BrainImaging + ECG + AMS + MentalStatus + Deliri + Hallucin + Confus + 
                        REorient + DISorient + Encephalopathy + UrineCulture + BloodCulture + LumbarPuncture + 
                        ABG + ElectrolytePanel + RenalFunction + CBC + LiverFunction +
                        ThyroidFunction + Cortisol + ToxScreen + AutoimmuneSerology + Bvitamins + 
                        Ammonia + SedimentationRate + HIVantibody + AntiPsychotics + Benzodiazepines + 
                        Dexmedetomidine + Rivastigmine,
                   data=n[,1:31],
                   lambda2=l2, model="logistic")

modell1l2 <- penalized(response=n$status, 
                   penalized= ~CXR + BrainImaging + ECG + AMS + MentalStatus + Deliri + Hallucin + Confus + 
                        REorient + DISorient + Encephalopathy + UrineCulture + BloodCulture + LumbarPuncture + 
                        ABG + ElectrolytePanel + RenalFunction + CBC + LiverFunction +
                        ThyroidFunction + Cortisol + ToxScreen + AutoimmuneSerology + Bvitamins + 
                        Ammonia + SedimentationRate + HIVantibody + AntiPsychotics + Benzodiazepines + 
                        Dexmedetomidine + Rivastigmine,
                   data=n[,1:31],
                   lambda1=l1, lambda2=l2, model="logistic")
```

### Table A.3 Logistic regression model for the binary classification of delirium from 31 clinical actions

Coefficients and confidence intervals are presented for odds. p-values < 0.000005 are represented as “0.00000.”

|  | CoefficientOdds | LowerBound95 | UpperBound95 | Z.Value | p.Value |
| --- | --- | --- | --- | --- | --- |
| (Intercept) | 0.034 | 0.031 | 0.037 | -81.087 | 0.00000 |
| CXR | 0.979 | 0.963 | 0.995 | -2.592 | 0.00955 |
| BrainImaging | 1.042 | 1.004 | 1.081 | 2.167 | 0.03023 |
| ECG | 1.011 | 0.987 | 1.034 | 0.906 | 0.36469 |
| AMS | 0.947 | 0.914 | 0.981 | -2.981 | 0.00288 |
| MentalStatus | 1.144 | 1.114 | 1.176 | 9.766 | 0.00000 |
| Deliri | 1.121 | 1.082 | 1.163 | 6.249 | 0.00000 |
| Hallucin | 1.252 | 1.161 | 1.351 | 5.820 | 0.00000 |
| Confus | 1.160 | 1.123 | 1.199 | 8.872 | 0.00000 |
| REorient | 0.863 | 0.807 | 0.923 | -4.270 | 0.00002 |
| DISorient | 1.104 | 1.015 | 1.200 | 2.322 | 0.02022 |
| Encephalopathy | 0.989 | 0.943 | 1.035 | -0.484 | 0.62857 |
| UrineCulture | 1.131 | 1.084 | 1.179 | 5.682 | 0.00000 |
| BloodCulture | 1.024 | 0.996 | 1.052 | 1.658 | 0.09732 |
| LumbarPuncture | 1.036 | 0.978 | 1.094 | 1.232 | 0.21807 |
| ABG | 0.978 | 0.972 | 0.984 | -7.097 | 0.00000 |
| ElectrolytePanel | 1.004 | 0.995 | 1.013 | 0.899 | 0.36869 |
| RenalFunction | 1.041 | 1.024 | 1.058 | 4.871 | 0.00000 |
| CBC | 0.965 | 0.952 | 0.978 | -5.186 | 0.00000 |
| LiverFunction | 1.009 | 0.996 | 1.022 | 1.422 | 0.15504 |
| ThyroidFunction | 1.122 | 1.058 | 1.189 | 3.872 | 0.00011 |
| Cortisol | 0.926 | 0.873 | 0.980 | -2.619 | 0.00881 |
| ToxScreen | 1.275 | 1.217 | 1.336 | 10.243 | 0.00000 |
| AutoimmuneSerology | 0.408 | 0.292 | 0.556 | -5.464 | 0.00000 |
| Bvitamins | 1.451 | 1.315 | 1.598 | 7.477 | 0.00000 |
| Ammonia | 1.070 | 0.968 | 1.173 | 1.390 | 0.16454 |
| SedimentationRate | 1.124 | 1.002 | 1.255 | 2.042 | 0.04117 |
| HIVantibody | 0.479 | 0.314 | 0.705 | -3.574 | 0.00035 |
| AntiPsychotics | 1.443 | 1.400 | 1.488 | 23.589 | 0.00000 |
| Benzodiazepines | 1.076 | 1.048 | 1.103 | 5.614 | 0.00000 |
| Dexmedetomidine | 1.432 | 1.260 | 1.626 | 5.513 | 0.00000 |
| Rivastigmine | 1.560 | 0.234 | 5.494 | 0.591 | 0.55419 |

### A.3 Sensitivity Tuning

We tuned log-odds cut-points for logistic regression as a binary classifier using 6 methods from the cutpointr R-package, including:

Maximize the Youden Index…

```
library(cutpointr)
dat <- data.frame(p = p, stat = n$status)
cp <- cutpointr(dat, p, stat,
                method=maximize_metric,
                metric=youden)
summary(cp)
```

Maximize sensitivity + specificity…

```
cp <- cutpointr(dat, p, stat,
                method=maximize_metric,
                metric=sum_sens_spec)
summary(cp)
```

Maximize accuracy…

```
cp <- cutpointr(dat, p, stat,
                method=maximize_metric,
                metric=accuracy)
summary(cp)
```

Minimize ROC (0,1)…

```
cp <- cutpointr(dat, p, stat,
                method=minimize_metric,
                metric=roc01)
summary(cp)
```

Maximize accuracy given minimum sensitivity…

```
cp <- cutpointr(dat, p, stat,
                method=maximize_metric,
                metric=acc_constrain)
summary(cp)
```

Maximize sensitivity given minimal specificity.

```
cp <- cutpointr(dat, p, stat,
                method=maximize_metric,
                metric=sens_constrain)
summary(cp)
```

### Table A.4 Cut-point, sensitivity, specificity, and accuracy of 6 methods for tuning a threshold for a binary logistic classifier.

| Method | Cut-point | Accuracy | Sensitivity | Specificity |
| --- | --- | --- | --- | --- |
| Youden Index | -2.722 | 0.726 | 0.804 | 0.719 |
| Maximize sensitivity + specificity | -2.722 | 0.726 | 0.804 | 0.719 |
| Maximize accuracy | 1.076 | 0.922 | 0.0519 | 0.9969 |
| Minimize dist to ROC(0,1) | -2.613 | 0.7648 | 0.7566 | 0.7656 |
| Maximize accuracy, constraining sensitivity | -1.953 | 0.879 | 0.5 | 0.912 |
| Maximize sensitivity, constraining specificity | -3.066 | 0.535 | 0.921 | 0.501 |

### A.4.1 Comparison Model 1: Puelle’s classifier of 8 words with high PPV

We implement Puelle’s model based on the “language of delirium” as a multivariate logistic regression model with 8 binary predictors. We generate binary predictors by reverting counts to presence/absence of a word during an admission, such that 1 or more occurrences of a given word is a 1 and no occurrences is a 0.

```
# revert count data to binary
n$AMS <- ifelse(n$AMS == 0, 0, 1)
n$MentalStatus <- ifelse(n$MentalStatus == 0, 0, 1)
n$Deliri <- ifelse(n$Deliri == 0, 0, 1)
n$Hallucin <- ifelse(n$Hallucin == 0, 0, 1)
n$Confus <- ifelse(n$Confus == 0, 0, 1)
n$REorient <- ifelse(n$REorient == 0, 0, 1)
n$DISorient <- ifelse(n$DISorient == 0, 0, 1)
n$Encephalopathy <- ifelse(n$Encephalopathy == 0, 0, 1)
```

```
# logistic model
load(file=file.path(paths$data, "Counts-Cohort-Training.Rda"))
n <- training
puelle <- glm(status ~ 
                 AMS + MentalStatus + Deliri + Hallucin + Confus + REorient + DISorient + Encephalopathy
       , data=n, family=binomial)
summary(puelle)
```

```
## 
## Call:
## glm(formula = status ~ AMS + MentalStatus + Deliri + Hallucin + 
##     Confus + REorient + DISorient + Encephalopathy, family = binomial, 
##     data = n)
## 
## Deviance Residuals: 
##     Min       1Q   Median       3Q      Max  
## -4.2743  -0.3707  -0.3241  -0.3241   2.5953  
## 
## Coefficients:
##                Estimate Std. Error  z value Pr(>|z|)    
## (Intercept)    -2.92021    0.02600 -112.319  < 2e-16 ***
## AMS            -0.06469    0.01772   -3.650 0.000262 ***
## MentalStatus    0.20314    0.01247   16.284  < 2e-16 ***
## Deliri          0.12919    0.01782    7.248 4.24e-13 ***
## Hallucin        0.38757    0.03776   10.265  < 2e-16 ***
## Confus          0.22988    0.01575   14.591  < 2e-16 ***
## REorient       -0.15708    0.03300   -4.761 1.93e-06 ***
## DISorient       0.18286    0.04079    4.483 7.38e-06 ***
## Encephalopathy -0.01398    0.02215   -0.631 0.528056    
## ---
## Signif. codes:  0 '***' 0.001 '**' 0.01 '*' 0.05 '.' 0.1 ' ' 1
## 
## (Dispersion parameter for binomial family taken to be 1)
## 
##     Null deviance: 20198  on 36405  degrees of freedom
## Residual deviance: 18360  on 36397  degrees of freedom
## AIC: 18378
## 
## Number of Fisher Scoring iterations: 5
```

### Supplementary Figure A.1. Probability density plot of 4 reclassification groups generated by Puelle’s classifier

### A.4.2 Comparison Model 2: Kim’s re-classification

We simply re-categorize by Kim et. al’s criteria using presence of delirium ICD-9 code *or* antipsychotics as the identifier for delirium.

1. ICD Positives have a positive ICD-9 code
2. Reclassified Positives have received antipsychotics but do not have an ICD-9 code for delirium
3. Double Negatives do not have either antipsychotics or an ICD-9 code
4. There are no Reclassified Negatives.

```
n$kim <- ifelse(n$status == 1, "ICDPositive",
                ifelse(n$AntiPsychotics > 0, "ReclassPositive", "DoubleNegative"))
```
